# Anti-stigma training and positive changes in mental illness stigma outcomes in medical students in ten countries: a mediation analysis on pathways via empathy development and anxiety reduction

**DOI:** 10.1007/s00127-022-02284-0

**Published:** 2022-04-22

**Authors:** Laura C. Potts, Ioannis Bakolis, Tanya Deb, Heidi Lempp, Tushar Vince, Yasmin Benbow, William Waugh, San Kim, Syed Raza, Claire Henderson, The INDIGO READ Study Group, Graham Thornicroft, Shuntaro Ando, Shinsuke Kondo, Kayo Ichihashi, Kiyoto Kasai, Sosei Yamaguchi, Asami Matsunaga, Yasutaka Ojio, Makoto Ogawa, Chiyo Fujii, Andrea Candelas, Laura Martín, Andrea Jiménez, Cristina Castañeda, Cecilia Hernández, Jesús de la Higuera, José Eduardo Muñoz-Negro, Mercedes Sola, Rocío García, José Miguel Gota, Juan Francisco Mula, Ana López, Amadeo Oria, Jorge A. Cervilla, Aguila Bono, Dolores Franco, Jaime Gómez, Carmen Jiménez, Remedios Dorado, Evelio Ingunza, Irene Márquez, Diego de la Vega, Pablo Gª-Cubillana, Uta Ouali, Lamia Jouini, Yosra Zgueb, Fethi Nacef, Megan Campbell, Dan Stein, Judit Harangozo, Andrea Acs, Tünde Bulyáki, Gyöngyi Szabó, Tunde Masseyferguson Ojo, A. Ogunwale, A. O. Sowunmi, S. S. Awhangansi, Deji Ogundapo, O. T. Sodiya, Babatunde Fadipe, Andrew T. Olagunju, Adebayo R. Erinfolami, Peter O. Ogunnubi, Catarina Cardoso Tomás, Miroslava Janoušková, Dzmitry Krupchanka, Simon Vasseur Bacle, Antoine Colliez, Deborah Sebbane, Amaury C. Mengin, Pierre Vidailhet, Cyril Cazals, Andrea Fiorillo, Gaia Sampogna, Micaela Savorani, Valeria Del Vecchio, Mario Luciano, Giuseppina Borriello, Benedetta Pocai, Patricia Neves Guimaraes, Antônio Prates Caldeira, Pedro Paulo Narciso de Avelar

**Affiliations:** 1 Department of Biostatistics and Health Informatics, Institute of Psychiatry, Psychology and Neuroscience, King’s College London, London, SE5 8AF UK; 2 South London and Maudsley NHS Foundation Trust, London, UK; 3 Centre for Rheumatic Diseases, King’s College London, London, UK; 4 GKT School of Medical Education, Faculty of Life Sciences and Medicine, King’s College London, London, UK; 5 Health Service and Population Research Department, Institute of Psychiatry, Psychology and Neuroscience, King’s College London, London, UK

**Keywords:** Stigma, Discrimination, Physician empathy, Medical students, Simulated patients, Intergroup contact

## Abstract

**Purpose:**

Studies of mental illness stigma reduction interventions have been criticised for failing to evaluate behavioural outcomes and mechanisms of action. This project evaluates training for medical students entitled ‘Responding to Experienced and Anticipated Discrimination’ (READ), developed to focus on skills in addition to attitudes and knowledge. We aimed to (i) evaluate the effectiveness of READ with respect to knowledge, attitudes, and clinical communication skills in responding to mental illness-related discrimination, and (ii) investigate whether its potential effectiveness was mediated via empathy or/and intergroup anxiety.

**Methods:**

This is an international multisite non-randomised pre- vs post-controlled study. Eligible medical students were currently undertaking their rotational training in psychiatry. Thirteen sites across ten countries (*n* = 570) were included in the final analysis.

**Results:**

READ was associated with positive changes in knowledge (mean difference 1.35; 95% CI 0.87 to 1.82), attitudes (mean difference − 2.50; 95% CI − 3.54 to − 1.46), skills (odds ratio 2.98; 95% CI 1.90 to 4.67), and simulated patient perceived empathy (mean difference 3.05; 95% CI 1.90 to 4.21). The associations of READ with knowledge, attitudes, and communication skills but not with simulated patient perceived empathy were partly mediated through student reported empathy and intergroup anxiety.

**Conclusion:**

This is the first study to identify mediating effects of reduced intergroup anxiety and increased empathy in an evaluation of anti-stigma training that includes behavioural measures in the form of communication skills and perceived empathy. It shows the importance of both mediators for all of knowledge, skills, and attitudes, and hence of targeting both in future interventions.

**Supplementary Information:**

The online version contains supplementary material available at 10.1007/s00127-022-02284-0.

## Introduction

Stigma and discrimination related to mental illness constitute a significant public health problem, leading to reduced help seeking and access to healthcare [1, 2], fewer opportunities for education and work [3, 4], increased co-morbidity [5], and mortality [6, 7]. Recent international mental health policies have highlighted the need for interventions to reduce discrimination [8, 9]. Several occupational groups have been identified as important sources of stigma and discrimination [10]. One is health-care staff, who was a target group for national anti-stigma campaigns in Canada [11] and Denmark [12] and a regional one in Andalucia [13].

On the other hand, a few studies have explored mental health professionals’ potential for leadership in reducing the impact of stigma and discrimination through training [14–16]. More broadly, training in health advocacy and social justice is provided to health professionals in some settings, most notably students and trainee primary care physicians [17, 18]. Training on stigma and discrimination therefore needs to acknowledge professionals’ as both sources of discrimination and as potential anti-stigma change agents.

To date, stigma education for medical students has focussed solely on stigma reduction [19, 20], with research showing short-term attitude changes. These projects have attended little to medical education research, which highlights critical reflection and self-reflection as ways to improve attitudes, beliefs, understanding of a subject, and satisfaction in learning [21]. Nor have such projects made use of skills training, which is widely evaluated in medical education via the observation of interactions based on standardised clinical presentations with simulated patients.

Meta-analysis of potential mediators of prejudice reduction through intergroup contact shows the importance of both increasing empathy for, and reducing anxiety about being with, the other group [22]. Among health-care professionals and students empathy is associated with patient satisfaction [23] and some clinical outcomes [24]. There has been little study of the role of these mediators in reducing negative attitudes towards people with mental illness despite the widespread use of contact interventions in this field [25]. It should not be assumed that their role is the same across groups; professionals with extensive occupational contact with people with mental illness are less likely to feel intergroup anxiety than are medical students. Understanding whether either has a mediating role in reducing negative attitudes within a given occupational or professional group will therefore inform further development of interventions that are tailored to the group to increase their potential effectiveness.

This project implements training for medical students entitled ‘Responding to Experienced and Anticipated Discrimination’ (READ), which applies the evidence bases from medical education and anti-stigma interventions and focusses on clinical skills in addition to attitudes and knowledge. READ aims to develop the role of future doctors to address and challenge mental illness-related discrimination, by improving medical students’ ability to: respond to discrimination, applying evidence for effective anti-stigma interventions; respond to anticipated discrimination and hence reduce loss of social and economic opportunities due to avoidance; and minimise behaviours that may be experienced by patients as discriminatory.

We aimed (i) to evaluate the effectiveness of READ for medical students by investigating potential changes in students’: knowledge, attitudes and skills in responding to mental illness-related discrimination and in simulated patients’ ratings of student empathy; (ii) to investigate whether the potential effectiveness of READ on the above outcomes was mediated via empathy or/and intergroup anxiety.

## Methods

### Study design and participants

This was an international multisite non-randomised pre- vs post-controlled study, including sites at 15 medical schools in 12 low-, middle-, and high-income countries (see Table S1 for sites and countries) [26]. The sites were either members of the INDIGO Network (a collaboration of researchers co-ordinated by the Centre for Global Mental Health, King’s College London) [27, 28] or received a personal invitation to the study. Eligible participants were medical students undertaking their rotational training in psychiatry of at least 1 week or taking classes in psychiatry, which takes place in different years of training in different countries. Participants were allocated to either READ or the control condition of usual teaching. Allocation of groups could not be randomised as delivery of READ was dependent on when this was feasible for trainers and the wider research team, who had no funding for the study; allocation of individuals to groups could not be randomised as this is determined by medical schools. The study was approved by King’s College London Psychiatry, Nursing and Midwifery Research Ethics Subcommittee (reference LRS-15/16-2894) and local approvals or exemptions were obtained at each site.

Sample size and power calculations have been described previously [26]. We calculated that 448 participants per group were required to achieve 90% power, accounting for 10% dropout, based on a standardised effect size of 0.66 on the MAKS.

### Intervention

READ was developed by members of the research team at King’s College London (CH, TD, and HL) in collaboration with people with lived experience: members of SUITE, the service user, carer, and family focussed part of the Education and Training Department of South London and Maudsley NHS Foundation Trust, and health professionals who were members of the Lived Experience Network, Oxleas Foundation Trust. Development included piloting with medical students at King’s College London who provided feedback via focus groups. The content, delivery, and evaluation of READ were informed by: (i) studies of patients’ experiences of discrimination [29]; (ii) research on stigma and discrimination among health-care professionals [10, 30, 31]; (iii) the literature on contact-based education to reduce stigma in health-care professionals [11, 32, 33] and students; and (iv) the broader field of study on intergroup contact to reduce prejudice [22, 34]. READ was designed to help students interact more effectively with patients they meet during the psychiatry rotation and hence enhance their overall learning rather than add unnecessarily to workload. READ was provided to small groups by the site research teams according to local teaching arrangements. The first session is delivered over 1.5 h near the start of the psychiatry rotation in each medical school; the second session, lasting 1 h, took place later before the end of the rotation. The length of the psychiatry rotation varies across medical schools; however, sites aimed to allow at least 1 week between the two sessions. This allowed time for students to identify the examples of discrimination to discuss in the second session. Further details on session content can be found in the study protocol [26].

READ was manualised and includes suggestions on adapting the training to each site’s resources and culture and the inclusion of testimonials by people with lived experience of mental illness, either in person or filmed. The sites also had the option to correspond with each other to share strategies and resources for delivery. A fidelity checklist was included with the manual, covering the facilitators for prejudice reduction identified by intergroup contact research [34] and studies with health-care professionals specifically [11]. Sites were encouraged to complete this for each session.

### Measures

The published versions of the scales in English were used at the sites in Nigeria, South Africa, and England. At all other sites, pre-existing published translations were used as available [35–41]. Where there was no pre-existing translation, site teams translated the scales following instructions from the INDIGO network (https://indigo-group.org/wp-content/uploads/2016/07/TranslationGuidelinesforIOPstigmascales-updated-18-08-16.pdf).

#### Mental health-related knowledge

Stigma-related knowledge was measured using the Mental Health Knowledge Schedule (MAKS) [42]. The MAKS comprises six items covering stigma-related mental health knowledge domains: help seeking, recognition, support, employment, treatment, and recovery; and six items regarding classification of various conditions as mental illnesses. The total score used the first six items and is calculated, so that higher MAKS scores indicate greater mental health knowledge. Previous work found that the scale has overall test–retest reliability of 0.71 (Lin’s concordance statistic) and overall internal consistency among items of 0.68 (Cronbach’s alpha) [42]. Validity is supported by extensive review by experts (including service users and international experts in stigma research).

#### Attitudes to mental illness

To measure medical students’ attitudes, we used the Mental Illness Clinician’s Attitudes (MICA2) scale [43]. A lower total score indicates less stigmatising attitudes to people with mental illness and to psychiatry as a medical speciality. This 16-item scale demonstrated good internal consistency (Cronbach’s alpha 0.79) and test–retest reliability (Lin’s concordance 0.80) [43]. Face and content validity were assessed through focus groups with medical students and the scale revised according to their suggestions; convergent and divergent validity were estimated, but require further assessment with larger samples. No relationship with scores on the Marlowe–Crowne Social Desirability Scale was found.

#### Behaviour and communication skills

Students’ behaviour and communication skills were assessed via an Objective Structured Clinical Examination (OSCE), where a student interacts with a simulated patient for a set period in the presence of an examiner [44]. This is a widely used assessment method in medical education, including for assessment of communication skills. The OSCE used in the study was developed by the research team at King’s College London including the Head of Clinical Assessment (TV), using the GKT School of Medicine’s template and marking scheme for all OSCEs used to assess medical students. We designed the scenario to fit the aims of the training. It required each medical student to discuss a referral to a local mental health team for treatment of the service user’s psychosis. The simulated patient, role played by either members of the research team or their clinical colleagues, reported experienced and anticipated discrimination. The sites were provided with the OSCE scenario and a standardised marking scheme describing the objectives and assessment process. The OSCE simulated patients were briefed in writing to help them standardise their role and responses to students. Examiners (members of the research team at each site) were given guidance on rating the outcome as either pass, borderline pass, borderline fail, or fail [45]. The student was expected to acknowledge and explore the service user’s experience and concerns and demonstrate empathy (verbally or nonverbally expressed). The student was assessed on (i) their response to the reports of anticipated and experienced discrimination; (ii) the extent to which they acknowledge the stereotypes of people with psychosis and distinguish these from the actual diagnosis and proposed treatment. At four sites, it was possible to mask examiners to participants’ group allocation. The interrater reliability among examiners could not be determined, because there was one examiner per site.

Simulated patients also assessed each student on empathic engagement using the 5-item JSPPPE (Jefferson Scale of Patient Perception of Physician Empathy) [46]. Results of the OSCE were provided to students at the end of the second training session in the form of individual oral feedback, as students in the pilot teaching sites indicated that this was valuable in improving their skills and exam performance.

#### Empathy

The Medical Student version of the Jefferson Scale for Empathy (JSE-S) is a 20-item scale with well-established psychometric properties [47, 48]. Each item has a 7-point Likert scale and the total score indicates greater empathy.

#### Intergroup anxiety

We employed Stephan and Stephan’s (SS-12) 12-item intergroup anxiety measure, modified for medical students [49]. The scale asks respondents to rate how much they experienced a range of feelings (anxious, apprehensive, comfortable, secure, worried, calm, confident, awkward, tense, carefree, nervous, and at ease) from 0 = not at all to 4 = extremely. A score is created by reverse scoring the positive feelings and averaging all the items, so that a higher score indicates greater anxiety. It has a reported internal consistency (Cronbach’s alpha) of 0.86; relationships with intergroup contact, stereotyping, and assumed dissimilarity support its construct validity.

### Study procedures

Informed one-to-one consent was taken at the start of the first session and an information sheet was provided at least 24 h prior. Once written informed consent was received, participating students in both the intervention and control group completed the self-report questionnaire-based measures followed by the OSCE. This was then repeated at the end of session 2, once the intervention group had completed the READ training [26].

Students were allocated by the trainer to either the control or the intervention group according to their usual local teaching group allocation at each site. Depending on what was feasible at each site, the control group completed the measures at the same time as the intervention group (five sites), or consecutive psychiatry rotations acted as the intervention and control groups.

### Statistical analysis

All analyses were done using Stata (version 16) and restricted to complete case analysis. Descriptive statistics for participant demographics and outcome and mediation measures (pre- and post-intervention) were calculated and reported by treatment group and overall. Continuous symmetric (non-skewed) measures were described using mean and standard deviation and categorical measures were described using both numbers and proportions (percentage). Baseline demographic, outcome, and mediation measures were also summarised by country.

Associations between READ training and MAKS, MICA2, JSPPPE, and OSCE scores in medical students were investigated by estimating the between group differences in each post-intervention measure of knowledge, attitudes, and skills (including patient perception of empathy). A complete case analysis was implemented with the use of random intercept generalised linear models to take into account the degree of clustering (individuals were clustered within countries) in our dataset. We included treatment group, baseline outcome measure, and age and gender of the participant as covariates in our models. Effect sizes (mean difference or odds ratio depending on the outcome) were presented with 95% confidence intervals.

#### Sensitivity analysis

We conducted three sensitivity analyses:

(i) To examine a different hierarchical structure, we re-ran our random intercept models with individuals being clustered within site rather than within country.
(ii) To explore the degree of heterogeneity across different countries, we ran a meta-analysis. Within each country, we used linear and ordinal regression models to investigate associations between READ training and MAKS, MICA2, OSCE, and JSPPPE. Regression results were pooled across countries using random effects meta‐analysis, and heterogeneity was summarised using the I-square statistic [50]. OSCE results from Italy were not included as they all contained the same result, “clear pass”.
(iii) To account for missing data, we imputed to a complete dataset using multiple imputation by chained equations (MICE) and re-ran our primary analysis models.

#### Mediation analysis

**Fig. 1:**
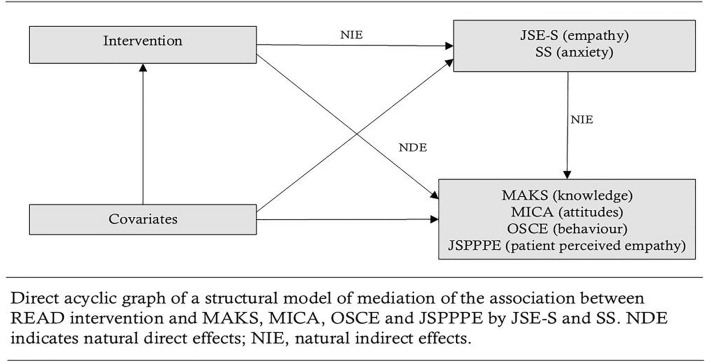
Mediating pathway of the association of READ intervention with MAKS, MICA, OSCE, and JSPPPE

To explicate the effect of READ training on knowledge, attitudes, skills, and patient perceived empathy, indirect associations acting through multiple continuous mediators’ (*intergroup anxiety and empathy* as mediating variables) and direct effects not mediated by *intergroup anxiety and empathy* and their standard errors were quantified (Fig. 1).

We tested the assumptions of our model [51] and investigated the effect of the READ intervention on each one of the multiple continuous mediators with the use of random intercept linear regression models taking into account the clustering within countries. We chose priori a *p* value of lower than 0.15 to select the appropriate mediators to include in our final mediation analysis. We tested the independence of the mediators by examining partial correlations between our mediators after accounting for treatment allocation.

A parametric multilevel linear regression mediation approach (*gsem* package in Stata version 16 [StataCorp]) was used to estimate the total effect, the natural indirect effects (NIE), and natural direct effects (NDE) of READ intervention on MAKS, MICA2, OSCE, and JSPPPE. The NDE represented the effect of READ intervention on knowledge, attitudes, skills, and patient perceived empathy that was independent of *intergroup anxiety and empathy*. An NIE represented the proportion of the relationship between the READ intervention and knowledge, attitudes, skills, and patient perceived empathy that could be explained by its effect with changes in intergroup anxiety and empathy. To quantify the magnitude of mediation, the study estimated the proportion of the effect mediated by *intergroup anxiety and empathy* (NIE/[NDE + NIE]). All analyses were estimated using bootstrapping (500 replications) to recover the correct SEs for direct and indirect effects. All models adjusted for the baseline outcome measure, and age and gender of the participant. All *p* values were two-tailed, and statistical significance was set at a *p* value less than 0.05.

## Results

### Recruitment

Out of 25 centres that initially agreed to participate in the READ study, 10 did not proceed to recruitment due to either lack of capacity of the site lead or inability to engage a medical school in the study. Between October 2016 and December 2019, 653 medical students undertaking their rotational training in Psychiatry from 15 centres amongst 12 countries consented to take part. Each site provided at minimum delivery of the intervention to one group of students. The sample size of 896 was not achieved due to the initial drop off in sites. Our original sample size calculation was based on 36 students recruited per site, which was achieved. Thirteen sites across ten countries (*n* = 570) were included in the final analysis, due to two sites not being able to run a comparator control group.

### Description of sample

Demographic, outcome, and mediation measures of participants are summarised in Table 1, overall and by intervention group. Summary statistics by country are presented in Supplementary Table S2.

**Table 1:** Demographic and outcome measures by intervention group

		Control (*n* = 275)				Intervention (*n* = 297)				Total (*n* = 572)			
		Pre		Post		Pre		Post		Pre		Post	
		*N*	*n* (%)/mean (SD)	*N*	*n* (%)/mean (SD)	*N*	*n* (%)/mean (SD)	*N*	*n* (%)/mean (SD)	*N*	*n* (%)/mean (SD)	*N*	*n* (%)/mean (SD)
Gender of student, *n* (%)	Female	274	161 (58.8)			296	177 (59.8)			570	338 (59.3)		
	Male		113 (41.2)				119 (40.2)				232 (40.7)		
Age of student, *n* (%)	< 22	255	66 (25.9)			297	49 (16.5)			552	115 (20.8)		
	22–24		143 (56.1)				191 (64.3)				334 (60.5)		
	25–27		31 (12.2)				44 (14.8)				75 (13.6)		
	28–30		9 (3.5)				10 (3.4)				19 (3.4)		
	> 30		6 (2.4)				3 (1.0)				9 (1.6)		
Year of student, *n* (%)	Early years	254	75 (29.5)			273	40 (14.7)			527	115 (21.8)		
	Late years		179 (70.5)				233 (85.3)				412 (78.2)		
OSCE result, *n* (%)	Clear fail	196	26 (13.3)	170	16 (9.4)	224	27 (12.1)	198	6 (3.0)	420	53 (12.6)	368	22 (6.0)
	Borderline fail		64 (32.7)		40 (23.5)		78 (34.8)		41 (20.7)		142 (33.8)		81 (22.0)
	Borderline pass		52 (26.5)		65 (38.2)		71 (31.7)		61 (30.8)		123 (29.3)		126 (34.2)
	Clear pass		54 (27.6)		49 (28.8)		48 (21.4)		90 (45.5)		102 (24.3)		139 (37.8)
JSPPPE total score, mean (SD)		217	20.3 (8.1)	198	21.3 (7.6)	246	19.7 (7.6)	236	24.0 (7.3)	463	20.0 (7.8)	434	22.8 (7.6)
MAKS total score, mean (SD)		258	20.8 (4.8)	244	21.9 (5.3)	291	21.4 (4.1)	283	23.6 (5.1)	549	21.1 (4.5)	527	22.8 (5.3)
MICA total score, mean (SD)		260	45.4 (9.9)	245	42.5 (10.7)	290	45.5 (11.1)	280	41.2 (13.0)	550	45.5 (10.6)	525	41.8 (12.0)
JSE-S total score, mean (SD)		234	104.7 (16.2)	241	107.5 (16.0)	266	105.3 (16.3)	280	109.5 (16.9)	500	105.0 (16.2)	525	108.6 (16.5)
SS total score, mean (SD)		261	2.0 (0.6)	245	1.7 (0.7)	294	2.0 (0.7)	284	1.5 (0.6)	555	2.0 (0.6)	529	1.6 (0.7)

The study sample comprised of a slightly higher proportion of females (60%) than males. The majority of students were aged 22–24 years (60%) and very few older than 28 years (5%). These findings were similar across treatment groups. Over three-quarters (78%) of the participating students were late year students, but this differed by treatment group, reducing to 70% in the control group and increasing to 85% in the intervention group.

#### Changes in students’ knowledge, attitudes, and skills in responding to mental illness-related discrimination

The results show that participants in the intervention group scored on average 1.35 (95% CI 0.87 to 1.82) points higher on the MAKS compared to those in the control group (Table 2), indicating greater mental health knowledge. Similarly, participants in the intervention group scored on average 2.5 (95% CI − 3.54 to − 1.46) points lower on the MICA2 than those in the control group, highlighting less stigmatising attitudes. The students in the intervention group also showed greater communication and behaviour skills as they had 2.98 (95% CI 1.90 to 4.67) times higher odds of receiving a higher category OSCE score than the control group students. The results also showed an increase in patient perception of empathy in the intervention group, as they scored on average 3.05 points (95% CI 1.90 to 4.21) higher on the JSPPPE than the control group.

**Table 2:** Associations between the READ intervention and mental health-related knowledge (MAKS), attitudes (MICA2), and skills (OSCE) in medical students

Predictors	MAKS^a^ (*n *= 501)	MICA^a^ (*n* = 500)	OSCE^b^ (*n* = 349)		JSPPPE^a^ (*n* = 413)
	MD (95% CI)	MD (95% CI)	OR (95% CI)		MD (95% CI)
Control (ref)	–	–	–		–
Intervention	1.35** (0.87, 1.82)	− 2.50** (− 3.54, − 1.46)	2.98** (1.90, 4.67)		3.05** (1.90, 4.21)
Baseline adjustment for outcome	0.84** (0.77, 0.09)	0.63** (0.56, 0.70)	Clear pass (ref)	–	0.44**(0.36, 0.53)
			Borderline pass	0.11** (0.04, 0.27)	
			Borderline fail	0.04** (0.01, 0.10)	
			Clear fail	0.01** (0.004, 0.04)	
Age					
< 22 (ref)	–	–	–		–
22–24	− 0.40 (− 1.10, 0.30)	1.23 (− 0.31, 2.78)	0.72 (0.42, 1.24)		− 0.11 (− 1.64, 1.42)
25–27	− 0.25 (− 1.18, 0.68)	2.83** (0.80, 4.86)	0.50 (0.22, 1.15)		− 1.83 (− 4.11, 0.45)
28–30	0.57 (− 0.94, 2.09)	4.13* (0.79, 7.47)	1.32 (0.29, 6.07)		− 0.6 (− 4.91, 3.70)
> 30	− 1.27 (− 3.14, 0.60)	− 0.87 (− 4.98, 3.24)	0.62 (0.09, 4.15)		1.67 (− 3.68, 7.02)
Gender					
Female (ref)	–	–	–		–
Male	− 0.09 (− 0.60, 0.42)	− 0.09 (− 1.22, 1.03)	0.95 (0.60, 1.49)		− 0.28 (− 1.52, 0.95)

^a^Multi-level mixed-effects linear regression where students are clustered within countries, with estimates of the mean difference (MD) presented

^b^Multi-level mixed-effects ordered logistic regression where students are clustered within countries, with estimates of the odds ratio (OR) presented

*p < 0.05

**p < 0.01

Our first sensitivity analysis, which adjusted for site rather than country as a random effect in the model, showed no difference in results. Our second sensitivity analysis, using meta-regression on country, also produced very similar effect estimates; however, high heterogeneity amongst sites was observed. Our final sensitivity analysis on a complete imputed dataset also showed no difference in results. These results can be found in Supplementary Table S3, S4 and Supplementary Fig. 1.

#### Pathways of empathy and intergroup anxiety on knowledge, attitudes, and skills to mental illness discrimination

Intergroup anxiety and student empathy were shown to be weakly correlated (correlation coefficient 0.18), so the decision was made to treat them as independent mediators. Table 3 presents the total, direct, and indirect effects of READ intervention on MAKS, MICA2, JSPPPE, and OSCE.

**Table 3:** Adjusted direct and indirect associations of knowledge (MAKS), attitudes (MICA), and skills (OSCE) with empathy (JSE-S) and anxiety (SS)

Measure	MAKS^a^ (*n* = 458^c^/503^d^)	MICA^a^ (*n* = 458^c^/503^d^)	OSCE^b^ (*n* = 340^c^/374^d^)	JSPPPE^b^ (*n* = 377^c^/418^d^)
	Estimate (95% CI^e^)	Estimate (95% CI^e^)	Estimate (95% CI^e^)	Estimate (95% CI^e^)
JSE-S				
Total association	**1.51 (1.06, 1.98)	**− 2.60 (− 3.59, − 1.52)	**3.04 (1.99, 5.19)	**3.05 (1.85, 4.24)
Direct association	**1.33 (0.91, 1.76)	**− 1.95 (− 2.93, − 0.97)	**2.87 (1.82, 4.52)	**3.16 (1.94, 4.38)
Indirect association via JSE-S	**0.17 (0.07, 0.31)	**− 0.64 (− 1.04, − 0.30)	**1.06 (0.98, 1.18)	− 0.12 (− 0.37, 0.13)
Proportion mediated (%)	**11.45 (4.31, 22.16)	**24.78 (12.38, 42.57)	8.38 (− 3.08, 22.94)	3.84 (− 4.53, 12.20)
SS				
Total association	**1.33 (0.86, 1.81)	**− 2.49 (− 3. 65, − 1.41)	**2.93 (1.92, 4. 93)	**3.10 (1.94, 4.25)
Direct association	**1.15 (0.68, 1.62)	**− 1.80 (− 2.80, − 0.79)	**2.74 (1.74, 4.34)	**3.29 (2.10, 4.49)
Indirect association via SS	**0.18 (0.07, 0.34)	**− 0.69 (− 1.13, − 0.35)	**1.07 (0.96, 1.24)	− 0.20 (− 0.50, 0.11)
Proportion mediated	**13.64 (5.47, 29.65)	**27.89 (13.71, 51.33)	9.37 (− 6.75, 29.32)	6.32 (− 3.77, 16.41)

Adjusted for age, sex, and baseline outcome/mediator

^a^Multi-level by country (random intercept) mixed-effects linear regression with estimates of the mean difference (MD) presented

^b^Multi-level by country (random intercept) mixed-effects ordered logistic regression with estimates of the odds ratio (OR) presented

^c^Number of observations in JSE-S model

^d^Number of observations in SS model

^e^95% percentile intervals from bootstrapping with 1000 replications

**p < 0.01

### Knowledge

The indirect effect via intergroup anxiety and empathy implied that we would, on average, observe a 0.18-point (95% CI 0.07 to 0.34) and 0.17-point (95% CI 0.07 to 0.31) increase in MAKS levels among participants who received the READ intervention through the mediator pathway. The proportions of the effect between the READ intervention and knowledge mediated by intergroup anxiety and empathy were 13.6% and 11.5%, respectively. The direct effect of READ indicated that we would, on average, observe a 1.15-point (95% CI 0.68 to 1.62) increase in the level of knowledge if all medical students were free of intergroup anxiety, and a 1.33-point (95% CI 0.91, 1.76) increase in the level of knowledge if all medical students were scoring the maximum for empathy.

### Attitudes

The indirect effects via intergroup anxiety and empathy implied that we would, on average, observe a 0.69-point (95% CI − 1.13 to − 0.35) and a 0.64-point (95% CI − 1.04 to − 0.30) decrease in MICA2 levels among participants who received the READ intervention through the mediator pathway. The proportions of the effect between the READ intervention and attitudes mediated by intergroup anxiety and empathy were 27.9% and 24.8%, respectively. The direct effect of READ indicated that we would, on average, observe a 1.80-point (95% CI − 2.80 to − 0.79) decrease in attitude level if all medical students were free of intergroup anxiety, and a 1.95-point (95% CI − 2.93 to − 0.97) decrease in attitude level if all medical students were scoring the maximum for empathy.

### Skills

The indirect effects via intergroup anxiety and empathy implied that we would, on average, observe a 1.07-odds (95% CI 0.96 to 1.24) and a 1.06-odds (95% CI 0.98 to 1.18) increase in OSCE levels among participants who received the READ intervention through the mediator pathway. The proportions of the effect between the READ intervention and skills mediated by intergroup anxiety and empathy were 9.4% and 8.4%, respectively. The direct effect of READ indicated that we would, on average, observe a 2.74-odds (95% CI 1.74 to 4.34) increase in skills level if all medical students were free of intergroup anxiety and a 2.87-odds (95% CI 1.82 to 4.52) increase in skills level if all medical students were scoring the maximum for empathy.

### Patient perceived empathy

The indirect effects via intergroup anxiety and empathy implied that we would, on average, observe a 0.20-point (95% CI − 0.50 to − 0.11) and a 0.12-point (95% CI − 0.37 to 0.13) decrease in JSPPPE levels among participants who received the READ intervention through the mediator pathway. The proportions of the effect between the READ intervention and patient perceived empathy mediated by intergroup anxiety and empathy were 6.3% and 3.8%, respectively. The direct effect of READ indicated that we would, on average, observe a 3.29-point (95% CI 2.10 to 4.49) increase in level of patient perceived empathy if all medical students were free of intergroup anxiety, and a 3.16-point (95% CI 1.94 to 4.38) increase in level of patient perceived empathy if all medical students were scoring the maximum for empathy.

## Discussion

In a large international multisite study, anti-stigma training was associated with positive changes in knowledge, attitudes, skills, and patient perceived empathy among medical students; this association was partly mediated through empathy development and intergroup anxiety. We found that the same mediators that had a positive effect on knowledge, attitudes, and skills had a potential negative effect on patient perceived empathy; we discuss this below. This is the first study that identifies mediating effects of increased intergroup contact and empathy in a study of the effects of an anti-stigma training intervention. As the mediation was partial, there is evidence for other mechanisms of effect, including direct effects and/or other mediators yet to be identified.

### Comparison with previous studies

Our findings are consistent with the literature on the mechanisms of action of intergroup contact [22]; both reduction in anxiety and increased empathy were important mediators of the observed improvement in attitudes to mental illness. This also applied, though to a lesser extent, to stigma-related knowledge, and to a small extent for the skills shown in the OSCE.

Our use of knowledge as an outcome differs much other research on intergroup contact. In a recent meta-analysis, knowledge was studied as a potential mediator and was not found to be as important as anxiety or empathy [22]. We used a stigma-related knowledge measure as an outcome instead for two main reasons. First, a public health conceptualisation of stigma implies evaluation of stigma reduction with respect to knowledge along with attitudes and behaviour, as recommended by the UK’s National Institute for Health and Care Excellence [52]. Second, health professionals and health-care students can be affected by a clinical bias due to exposure to people with mental illness during crisis and to people with more long-term and disabling illness. This can lead to therapeutic pessimism, which for this target group is an important target outcome, and is addressed by several MAKS items. We have thus tailored the outcomes to the mental health-care context [53]. Our results suggest that reduced anxiety and greater empathy can promote more therapeutic optimism.

The smaller mediating effects of intergroup anxiety and empathy on the behaviours demonstrated in the OSCE support the view that while these effects are useful in promoting these behaviours, their ability to predict behaviour in practice is precarious. Literature shows that while communication skills teaching can improve empathy, the effects are sustained through continued clinical practice [54].

The lack of mediation by self-reported anxiety or empathy on simulated patient perceived empathy in the presence of a direct effect on skills highlights two key issues in professional education. One is the importance of using patient feedback in addition to observer ratings in the assessment of clinical skills; and the other is the need to further explore the relationship between self-rated and patient-rated empathy. We found no relationship between the two in our data; on searching for other studies, we found one recent report of a negative correlation between the two, by authors who had found no previous studies [55]. These authors interpreted their results as suggesting that some physicians underestimate their level of empathy, raising concerns about the validity of this measure. Alternatively, the READ training may have been sufficient to improve performance in that students responded to the simulated patient’s concerns, but not in a way that was perceived as empathetic. Perceived empathy might improve with further clinical experience.

High heterogeneity of effect was observed across countries. This may be due to how the intervention was delivered; however, fidelity checklist scores showed little variation which meant that we could not explore this quantitatively. Another possible explanation is unmeasured student differences among the sites; contact-based education may be counterproductive for some students, although the evidence for this comes from a younger target group [56].

### Strengths and limitations

Research on anti-stigma interventions in mental health has been criticised for focussing almost exclusively on attitudes as an outcome [57]. In this study, we took advantage of OSCEs as a long-established and widely used assessment method used in medical education to measure students’ skills in addressing anticipated and experienced discrimination on the part of people with mental health problems. However, we cannot extrapolate the OSCE results directly to clinical practice, especially in settings where appointment times are very short and, therefore, the ability to replicate such discussions would be more constrained. Furthermore, medical students in some sites were more familiar with this method than at others, which may have led to variation in their performance both pre- and post-training. We also note that it was only possible to mask OSCE observers to allocation at four sites, and two sites could not administer the OSCE.

We also address a criticism of the evaluation of many contact-based interventions for mental health stigma reduction, namely lack of attention to mechanisms of action [25], through our measurement of the key mediators identified as reducing prejudice [22].

Our inclusion of a control group strengthened the design and allowed for the mediation analysis; however, the lack of any funding for this study meant that we needed to be pragmatic and we were unable to randomise individual students or groups of students, reducing our ability to infer causality.

Running the study in multiple countries and amongst multiple ethnicities increased generalisability. It is highly possible that different sampling frames were used in different countries. Both sporadic and systematic missing data were observed across all countries and the primary analysis was complete case. However, a sensitivity analysis imputing to a full dataset using multiple imputation by chained equations showed no difference in results.

The intervention both discusses mental illness in general and specific diagnoses in relation to stereotypes and those illnesses disclosed by people in the video clips used (schizoaffective disorder and bipolar disorder) and other disorders disclosed by the experts by experience who presented in person. However, some disorders were not covered, including substance use disorders and dementia. The results may therefore not be generalisable to these disorders, either in terms of the outcomes or the mediators.

### Implications for research and practice

There are two implications for medical education: regarding READ, and regarding the potential of psychiatry rotations to increase or reduce stigma. Our results suggest that READ can be used to improve communication skills in relation to addressing patients’ experiences of and anticipation of discrimination, which may be experienced as empathetic on the part of service users, something critical to the development of therapeutic relationships. The partial mediation by anxiety and empathy suggest that READ is effective in improving mental illness stigma-related knowledge, attitudes, and skills even among students who are empathetic and do not feel anxious about working with people with mental illness. Our OSCE scenario of referral from primary care to mental health services is important given the impact of stigma on help-seeking for people with severe mental illness [58], and the large proportion of medical students who pursue careers in primary care. Further research and modification to the content would be needed to examine whether this type of training can promote communication to reduce the impact of stigma by doctors in situations other than that covered by the OSCE. For example, doctors frequently have discussions with: patients concerned about disclosure of a mental health problem to a current or prospective employer [59]; stigmatising colleagues [60]; and with family members concerned about stigma affecting the patient and the wider family or who may themselves stigmatise the patient [29].

Psychiatry rotation leads have educational priorities beyond stigma reduction, but must consider the overall impact of students’ direct contact with service users and extended contact through contact with carers and mental health professionals, which may increase or decrease stigma [61]. Our results suggest that education and contact which reduce student anxiety about working with people with mental illness and preserving and promoting their empathy are both important in achieving stigma reduction during the rotation, and that this may facilitate therapeutic relationships and hence better outcomes in settings such as primary care.

## Supplementary Information

Below is the link to the electronic supplementary material.

Supplementary file1 (PDF 409 KB)
