# Supplementary material for: Anti-stigma training and positive changes in mental illness stigma outcomes in medical students in ten countries: a mediation analysis on pathways via empathy development and anxiety reduction
Source: Soc Psychiatry Psychiatr Epidemiol. 2022 Apr 22;57(9):1861–73. doi: 10.1007/s00127-022-02284-0 (PMC9375761; doi:10.1007/s00127-022-02284-0)

Anti-stigma training and positive changes in mental health related empathy development, anxiety reduction and stigma outcomes in medical students in ten countries: A mediation analysis

Laura C Potts[a], Ioannis Bakolis[b,j] Tanya Deb[c], Heidi Lempp[d], Tushar Vince[e], Yasmin Benbow[f], William Waugh[g], San Kim[h], Syed Raza[i], Claire Henderson[j] and the INDIGO READ study group*

[a]Department of Biostatistics and Health Informatics, Institute of Psychiatry, Psychology and Neuroscience, King's College London, London, UK. Email: laura.potts@kcl.ac.uk

[b]Department of Biostatistics and Health Informatics, Institute of Psychiatry, Psychology and Neuroscience, King's College London, London, UK. Email: ioannis.bakolis@kcl.ac.uk

[c]South London and Maudsley NHS Foundation Trust, London, UK. Email: tanya.deb@slam.nhs.uk

[d]Centre for Rheumatic Diseases, King's College London, London, UK. Email: heidi.lempp@kcl.ac.uk

[e]GKT School of Medical Education, Faculty of Life Sciences & Medicine, King's College London, UK. Email Tushar.vince@kcl.ac.uk

[f]Health Service and Population Research Department, Institute of Psychiatry, Psychology and Neuroscience, King's College London, UK.. Email: yasmin.benbow@btinternet.com

[g]GKT School of Medical Education, Faculty of Life Sciences & Medicine, King's College London, UK. William.waugh@nhs.net

[h]GKT School of Medical Education, Faculty of Life Sciences & Medicine, King's College London, UK. Email: sankim0123@gmail.com

[i]GKT School of Medical Education, Faculty of Life Sciences & Medicine, King's College London, UK. Syedahmer.raza@nhs.net

[j]Health Service and Population Research Department, Institute of Psychiatry, Psychology and Neuroscience, King's College London, UK. Email: claire.1.henderson@kcl.ac.uk

***INDIGO READ study group members**

Graham Thornicroft (King's College London), Shuntaro Ando, Shinsuke Kondo, Kayo Ichihashi, Kiyoto Kasai (The University of Tokyo Hospital, Japan), Sosei Yamaguchi, Asami Matsunaga, Yasutaka Ojio, Makoto Ogawa, Chiyo Fujii (National Institute of Mental Health, National Centre of Neurology and Psychiatry, Japan), Andrea Candelas, Laura Martín, Andrea Jiménez, Cristina Castañeda, Cecilia Hernández & Jesús de la Higuera (University Hospital of Puerto Real, Cadiz, Andalusia, Spain), José Eduardo Muñoz-Negro, Mercedes Sola, Rocío García, José Miguel Gota, Juan Francisco Mula, Ana López, Amadeo Oria, Jorge A. Cervilla & Aguila Bono (University Hospitals of Granada & Andalusian School of Public Health, Andalusia, Spain), Dolores Franco, Jaime Gómez, Carmen Jiménez, Remedios Dorado, Evelio Ingunza, Irene Márquez, Diego de la Vega & Pablo Gª-Cubillana (University Hospitals of Seville, Andalusia, Spain), Uta Ouali, Lamia Jouini, Yosra Zgueb, Fethi Nacef (Razi University Hospital, Tunisia), Megan Campbell, Dan Stein (University of Cape Town, South Africa), Judit Harangozo, Andrea Acs, Tünde Bulyáki, Gyöngyi Szabó (Semmelweis University of Medicine, Centre of Community Psychiatry, Budapest, Hungary), Tunde Masseyferguson Ojo, A. Ogunwale, A.O Sowunmi , S. S Awhangansi, Deji Ogundapo, O.T Sodiya, (Neuropsychiatric Hospital, Aro, Abeokuta, Nigeria), Babatunde Fadipe, Andrew T. Olagunju, Adebayo R. Erinfolami, Peter O. Ogunnubi (Lagos University Teaching Hospital, Idi-Araba, Lagos State, Nigeria), Catarina Cardoso Tomás, (School of Health Sciences of Polytechnic Institute of Leiria, Portugal), Miroslava Janoušková, Dzmitry Krupchanka (National Institute of Mental Health, Klecany, Czech Republic), Simon Vasseur Bacle (Centre Collaborateur OMS, CCOMS, Lille), Antoine Colliez, Deborah Sebbane (University of Lille 2, France), Amaury C. Mengin, Pierre Vidailhet, Cyril Cazals (UNISIMES, University of Strasbourg, France), Andrea Fiorillo, Gaia Sampogna, Micaela Savorani, Valeria Del Vecchio, Mario Luciano, Giuseppina Borriello, Benedetta Pocai (Department of Psychiatry, University of Campania "Luigi Vanvitelli", Naples, Italy), Patricia Neves Guimaraes, Antônio Prates Caldeira, Pedro Paulo Narciso de Avelar, Department of Mental and Public Health, State University of Montes Claros, Brazil.

Corresponding author: Laura C Potts. Department of Biostatistics and Health Informatics, Institute of Psychiatry, Psychology and Neuroscience, King's College London, London, SE5 8AF, UK. Email: laura.potts@kcl.ac.uk. ORCID ID: 0000-0002-2935-6532

Table S1: Distribution of sites for the READ study

	**Continent**	**Country**	**Number of sites**
	Europe	UK	1
		Czech Republic	1
		France	2
		Spain	2
		Italy	1
		Portugal	1
		Hungary	1
	South America	Brazil	1
	Asia	Japan	1
	Africa	South Africa	1
		Nigeria	2
		Tunisia	1
**Summary**	4	12	15

Table S2: Baseline (pre intervention) demographics and outcome measures by country

		UK (n=31)	Hungary (n=36)	Spain (n=97)	Czech Republic (n=55)	Japan (n=39)	Nigeria (n=85)	Portugal (n=36)	France (n=51)	Italy (n=60)	Tunisia (n=82)	Total (n=572)
Gender of student *n (%)*	female	13 (41.9)	8 (22.2)	64 (66.0)	33 (60.0)	7 (17.9)	46 (55.4)	27 (75.0)	32 (62.7)	49 (81.7)	59 (72.0)	338 (59.3)
	male	18 (58.1)	28 (77.8)	33 (34.0)	22 (40.0)	32 (82.1)	37 (44.6)	9 (25.0)	19 (37.3)	11 (18.3)	23 (28.0)	232 (40.7)
Age of student *n (%)*	<22	21 (67.7)	15 (41.7)	31 (32.3)	0 (0.0)	1 (2.6)	13 (16.3)	0 (0.0)	2 (3.9)	31 (66.0)	1 (1.2)	115 (20.8)
	22-24	9 (29.0)	17 (47.2)	50 (52.1)	40 (72.7)	34 (87.2)	45 (56.3)	11 (30.6)	36 (70.6)	15 (31.9)	77 (95.1)	334 (60.5)
	25-27	1 (3.2)	4 (11.1)	11 (11.5)	14 (25.5)	1 (2.6)	17 (21.3)	14 (38.9)	10 (19.6)	0 (0.0)	3 (3.7)	75 (13.6)
	28-30	0 (0.0)	0 (0.0)	2 (2.1)	0 (0.0)	2 (5.1)	5 (6.3)	8 (22.2)	1 (2.0)	1 (2.1)	0 (0.0)	19 (3.4)
	>30	0 (0.0)	0 (0.0)	2 (2.1)	1 (1.8)	1 (2.6)	0 (0.0)	3 (8.3)	2 (3.9)	0 (0.0)	0 (0.0)	9 (1.6)
Year of student *n (%)*	early years	31 (100.0)	0 (.)	0 (0.0)	0 (0.0)	0 (0.0)	0 (0.0)	24 (66.7)	0 (0.0)	60 (100.0)	0 (0.0)	115 (21.8)
	late years	0 (0.0)	0 (.)	97 (100.0)	55 (100.0)	39 (100.0)	76 (100.0)	12 (33.3)	51 (100.0)	0 (0.0)	82 (100.0)	412 (78.2)
OSCE result *n (%)*	Clear fail	2 (6.5)	0 (0.0)	22 (22.7)	0 (.)	6 (15.4)	11 (13.1)	0 (.)	7 (15.2)	0 (0.0)	5 (12.5)	53 (12.6)
	Borderline fail	23 (74.2)	5 (13.9)	40 (41.2)	0 (.)	18 (46.2)	23 (27.4)	0 (.)	17 (37.0)	0 (0.0)	16 (40.0)	142 (33.8)
	Borderline pass	6 (19.4)	15 (41.7)	25 (25.8)	0 (.)	12 (30.8)	32 (38.1)	0 (.)	14 (30.4)	1 (2.1)	18 (45.0)	123 (29.3)
	Clear pass	0 (0.0)	16 (44.4)	10 (10.3)	0 (.)	3 (7.7)	18 (21.4)	0 (.)	8 (17.4)	46 (97.9)	1 (2.5)	102 (24.3)
JSPPPE total score *Mean (sd)*		24.6 (1.5)	25.9 (7.1)	15.9 (7.4)	. (.)	18.2 (5.5)	17.0 (7.2)	. (.)	23.0 (5.6)	29.6 (2.4)	16.7 (7.2)	20.0 (7.8)
MAKS total score *Mean (sd)*		22.5 (2.1)	23.1 (2.2)	22.4 (3.4)	22.5 (2.1)	21.4 (2.2)	18.2 (6.4)	24.7 (2.1)	23.6 (2.2)	13.3 (2.1)	21.2 (2.5)	21.1 (4.5)
MICA total score *Mean (sd)*		35.8 (2.4)	39.1 (8.0)	39.1 (7.8)	58.4 (5.5)	59.3 (4.8)	53.1 (8.0)	33.5 (5.0)	42.0 (6.7)	44.9 (6.5)	44.8 (8.3)	45.5 (10.6)

	UK (n=31)	Hungary (n=36)	Spain (n=97)	Czech Republic (n=55)	Japan (n=39)	Nigeria (n=85)	Portugal (n=36)	France (n=51)	Italy (n=60)	Tunisia (n=82)	Total (n=572)
JSE-S total score *Mean (sd)*	113.7 (3.7)	111.9 (12.8)	118.5 (7.8)	84.5 (5.5)	82.8 (5.9)	105.7 (12.0)	119.3 (8.9)	107.9 (9.7)	108.7 (9.0)	94.0 (17.0)	105.0 (16.2)
SS total score *Mean (sd)*	1.2 (0.5)	1.7 (0.6)	1.9 (0.7)	2.2 (0.6)	2.6 (0.6)	2.2 (0.6)	2.0 (0.2)	1.8 (0.6)	1.7 (0.3)	2.1 (0.6)	2.0 (0.6)

Table S3: Associations between the READ intervention and mental health-related knowledge, attitudes and behaviour in Medical Students.

**Predictors**	**MAKS[¶] (n=501)**	**MICA[¶] (n=500)**	**OSCE† (n=349)**		**JSPPPE[¶] (n=)**
	**MD (95% CI)**	**MD (95% CI)**	**OR (95% CI)**		**MD (95% CI)**
Control (ref)	-	-		-	-
Intervention	1.28** (0.86, 1.70)	-2.35** (-3.34, -1.36)		2.95** (1.88, 4.62)	3.06** (1.90, 4.22)
Baseline adjustment for outcome	0.53** (0.45, 0.61)	0.59** (0.52, 0.66)	Clear pass (ref)	-	0.44** (0.35, 0.53)
			Borderline pass	0.11** (0.04, 0.28)	
			Borderline fail	0.04** (0.01, 0.10)	
			Clear fail	0.01** (0.004, 0.04)	
Age					
<22 (ref)	-	-		-	-
22-24	0.00 (-0.68, 0.68)	0.77 (-0.84, 2.38)		0.74 (0.42, 1.32)	-0.37 (-2.01, 1.27)
25-27	0.08 (-0.79, 0.96)	2.31* (0.24, 4.37)		0.49 (0.21, 1.17)	-2.22 (-4.58, 0.15)
28-30	0.23 (-1.13, 1.59)	4.36** (1.14, 7.58)		1.32 (0.29, 6.08)	-0.56 (-4.88, 3.76)
>30	-0.46 (-2.17, 1.24)	-1.23 (-5.27, 2.80)		0.56 (0.08, 3.79)	0.93 (-4.52, 6.39)
Gender					
Female (ref)	-	-		-	-
Male	-0.17 (-0.63, 0.29)	0.09 (-0.99, 1.17)		0.98 (0.62, 1.55)	-0.14 (-1.38, 1.11)

[¶]Multi-level mixed-effects linear regression where students are clustered within site, with estimates of the mean difference (MD) presented.

[†]Multi-level mixed-effects ordered logistic regression where students are clustered within site, with estimates of the odds ratio (OR) presented.

*p<0.05

**p<0.01

Table S4: Associations between the READ intervention and mental health-related knowledge, attitudes and behaviour in Medical Students on a fully imputed dataset by MICE.

Predictors	MAKS[¶] (n=572)	MICA[¶] (n=572)	OSCE† (n=572)		JSPPPE[¶] (n=572)
	MD (95% CI)	MD (95% CI)	OR (95% CI)		MD (95% CI)
Control (ref)	-	-		-	-
Intervention	1.31** (0.81, 1.80)	-2.53** (-3.57, -1.48)		3.02** (2.04, 4.47)	2.87** (1.71, 4.03)
Baseline adjustment for outcome	0.88** (0.81, 0.95)	0.66** (0.58, 0.73)	Clear pass (ref)	-	0.48** (0.40, 0.56)
			Borderline pass	0.16** (0.06, 0.40)	
			Borderline fail	0.04** (0.02, 0.10)	
			Clear fail	0.02** (0.006, 0.05)	
Age					
<22 (ref)	-	-		-	-
22-24	-0.32 (-1.02, 0.39)	1.61 (-0.08, 3.31)		0.72 (0.42, 1.23)	0.03 (-1.42, 1.49)
25-27	-0.32 (-1.27, 0.63)	3.05** (0.90, 5.20)		0.56 (0.25, 1.24)	-1.65 (-4.05, 0.76)
28-30	0.59 (-0.94, 2.13)	4.41** (1.27, 7.56)		1.15 (0.32, 4.15)	-0.45 (-4.28, 3.39)
>30	-1.36 (-3.27, 0.54)	-0.30 (-4.50, 3.89)		0.96 (0.13, 7.33)	1.53 (-4.61, 7.68)
Gender					
Female (ref)	-	-		-	-
Male	0.05 (-0.47, 0.58)	0.03 (-1.18, 1.25)		0.88 (0.52, 1.48)	-0.19 (-1.75, 1.37)

[¶]Multi-level mixed-effects linear regression where students are clustered within countries, with estimates of the mean difference (MD) presented.

[†]Multi-level mixed-effects ordered logistic regression where students are clustered within countries, with estimates of the odds ratio (OR) presented.

*p<0.05

**p<0.01

Figure S1: Forest plots on the meta-regression of the READ intervention on a) MAKS b) MICA2 c) OSCE and d) JSPPPE by country.

Figure S1a

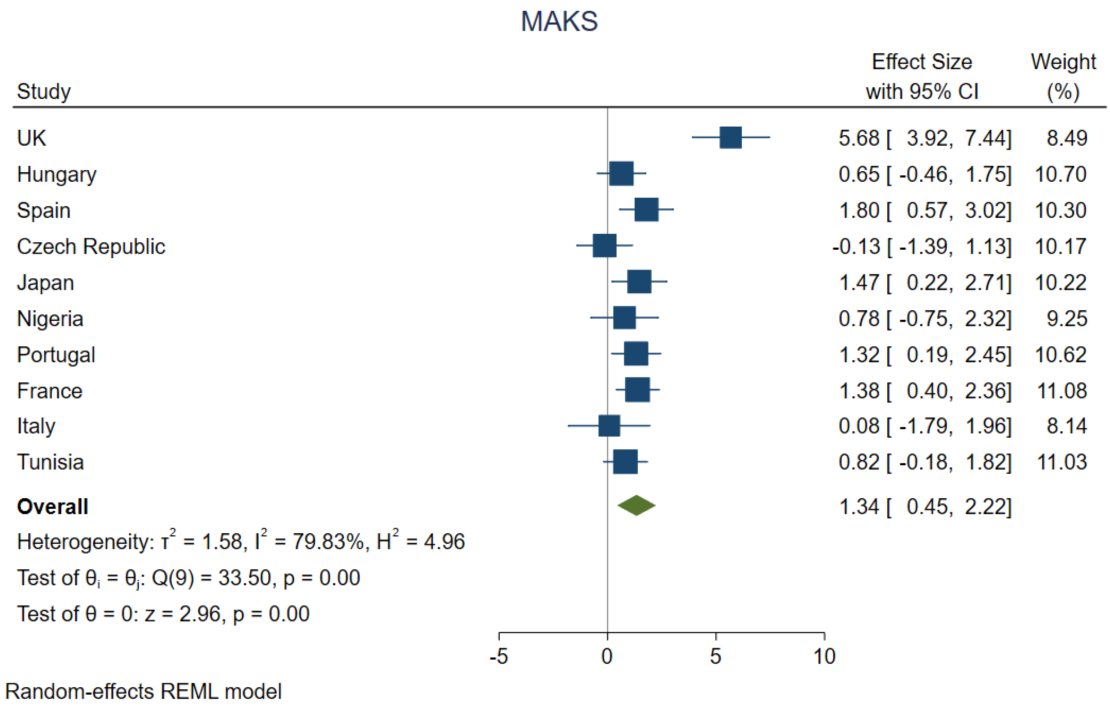

Figure S1b

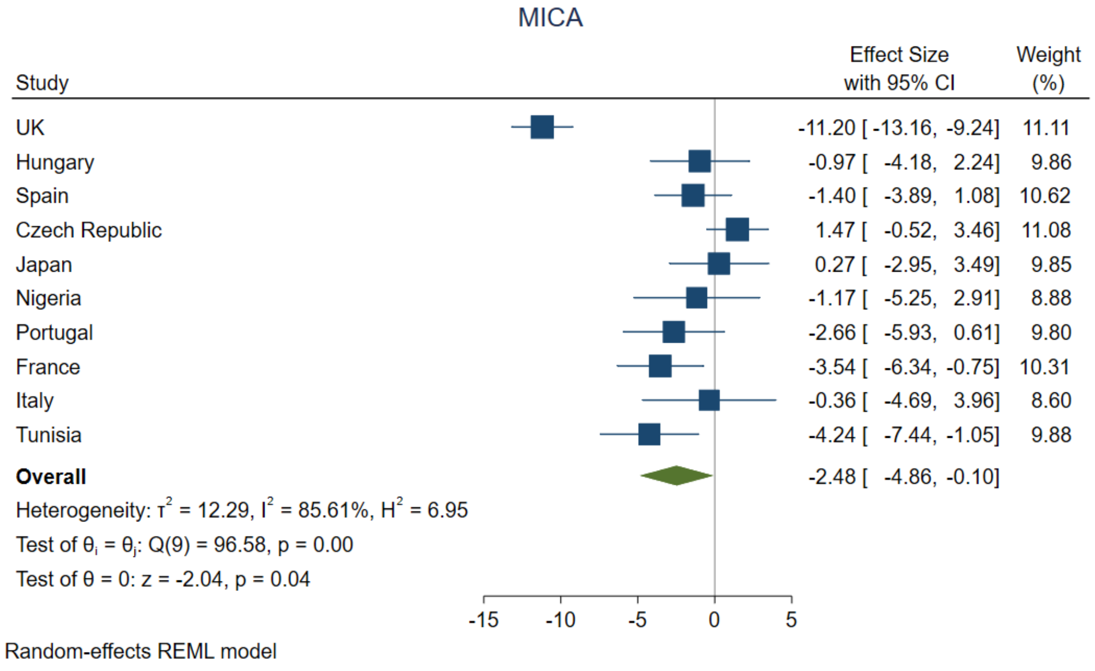

Figure S1c

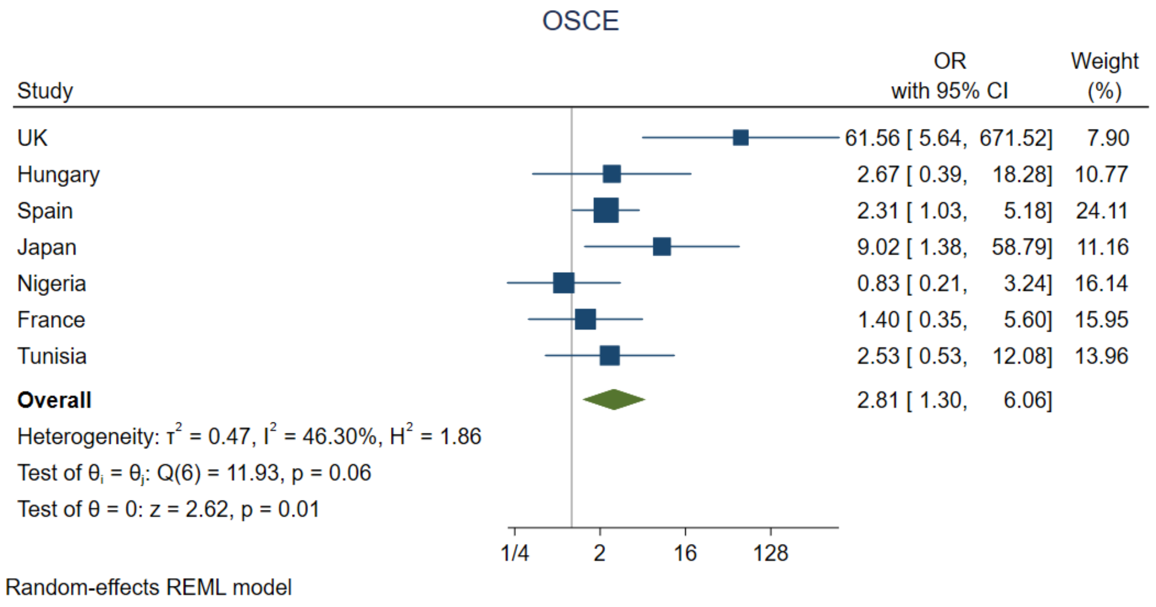

Figure S1d

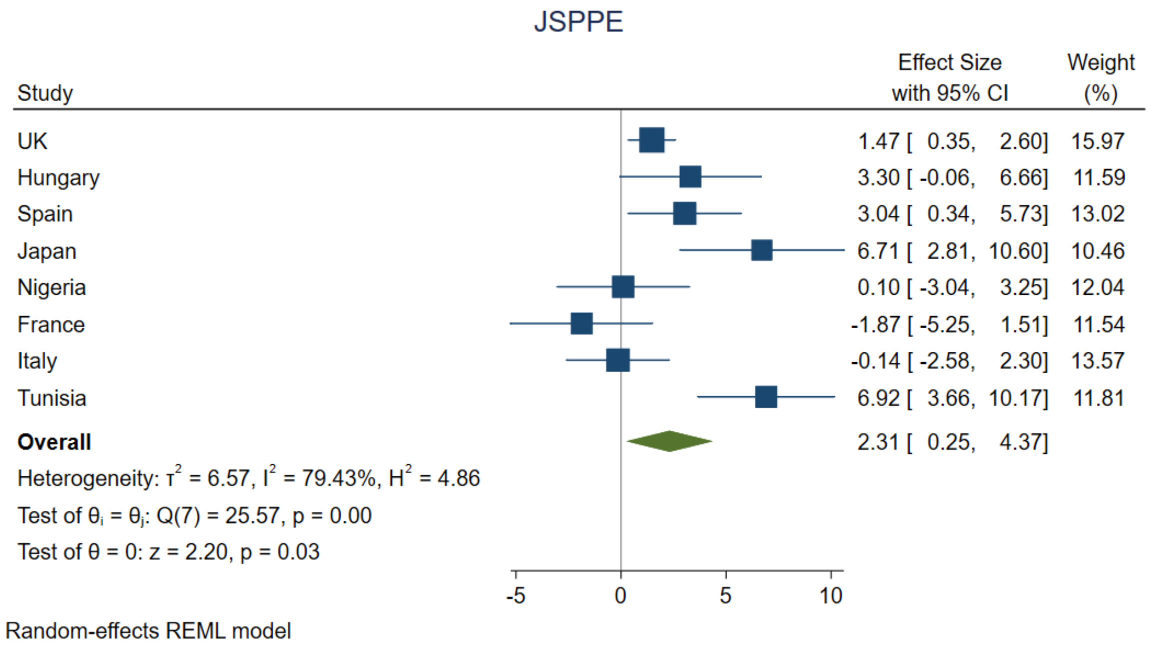

Supplement: Supplementary file 1 — Supplementary file1 (PDF 409 KB) [file 127_2022_2284_MOESM1_ESM.pdf]
